# Developmental outcomes for survivors of placental laser photocoagulation for the management of twin-to-twin transfusion syndrome

**DOI:** 10.1186/s12884-023-05997-5

**Published:** 2023-09-28

**Authors:** Angela Guzys, Susan M. Reid, Christie Bolch, Dinah S. Reddihough, Mark Teoh, Ricardo Palma-Dias, Alison Fung, Stephen Cole, Ryan Hodges, Michael Fahey, Susan P. Walker

**Affiliations:** 1 Neurodisability and Rehabilitation, Murdoch Children’s Research Institute, Melbourne, Australia; 2 Department of Paediatrics, University of Melbourne, Melbourne, Australia; 3 Department of Neurodevelopment and Disability, The Royal Children’s Hospital, Melbourne, Australia; 4 Fetal Diagnostics Unit, Monash Health, Melbourne, Australia; 5 Victorian Fetal Therapy Service, Monash Health, Melbourne, Australia; 6 Department of Obstetrics and Gynaecology, University of Melbourne, Melbourne, Australia; 7 Ultrasound Services, The Royal Women’s Hospital, Melbourne, Australia; 8 Mercy Perinatal, Mercy Hospital for Women, Melbourne, Australia; 9 Division of Maternity Services, The Royal Women’s Hospital, Melbourne, Australia; 10 Women’s and Newborn Program, Monash Health, Melbourne, Australia; 11 Paediatric Neurology Unit, Monash Children’s Hospital, Melbourne, Australia; 12 Department of Paediatrics, Monash University, Melbourne, Australia

**Keywords:** Fetal therapies, Fetoscopy, Twin-twin transfusion, Laser therapy, Twins

## Abstract

**Background:**

Fetoscopic laser coagulation of placental anastomoses reverses the pathological process in twin-to-twin transfusion syndrome, thereby increasing survival, but there are a paucity of studies addressing long-term neurodevelopmental outcome of survivors. This study aimed to ascertain the presence of neurodevelopmental disabilities in child survivors of monochorionic pregnancies managed by placental laser photocoagulation in the Australian state of Victoria.

**Methods:**

All pregnancies undergoing placental laser photocoagulation with the Victorian Fetal Therapy Service between 2006–2017 were included. Information on each surviving child, including demographics, perinatal course, and developmental progress was collected from parents, and consent was sought to complete the Child Behaviour Checklist. Interviewers evaluated whether this information was consistent with a diagnosis of any of 14 neurodevelopmental conditions. A three-tiered outcome measure was allocated for each child: (1) unimpaired or developmentally normal, (2) mild or moderate neurological impairment, or (3) severe neurological impairment. Clinical predictors for adverse outcome were identified.

**Results:**

Of 116 pregnancies (113 twin, 3 triplet), 96 (83%) resulted in 1 + surviving fetuses. 57/113 (50%) twin pregnancies resulted in 2 survivors, 36 (32%) in 1 survivor, and 20 (18%) in no survivors. Of the 235 fetuses, 154 (65.5%) survived to follow-up. Survival increased from 59% in 2006–2008 to 73% in 2015–2017. 90/154 (58%) survivors were followed up at a mean age of 7.5 [SD 3.0] years. Based on parental interview and Child Behaviour Checklist data, 28/90 (31%) participants were assessed as having neurodevelopmental impairment, 27 of mild-moderate severity and 1 severe. Speech/language disorders, attention deficit (hyperactivity) disorders, and fine motor impairment were most common. Neonatal length of stay conferred the highest risk of impairment.

**Conclusion:**

Substantial variation exists between fetal therapy services in the type and length of neonatal follow-up following fetoscopic laser coagulation, contributing to a lack of data on long-term outcomes. The findings from this study support increasingly urgent calls to undertake systematic and sustained follow-up of fetoscopic laser coagulation survivors until school age. Information from this study may assist parents in their decision-making when offered fetal surgery. Importantly, it highlights a group for targeted surveillance and early intervention.

## Introduction

Occurring spontaneously in 1 in 250 pregnancies, monozygotic twins arise from a single zygote and are therefore genetically identical [1]. In approximately 75% of cases, the developing fetuses share a single placenta following a split that occurs between days 4 and 8 post conception. Compared to singletons, monochorionic twins have a 3 to 7 times increased risk of perinatal morbidity and mortality [2], and they are 9 times more likely than dichorionic twins to die in utero [3]. This increased mortality and morbidity in monochorionic twins may be attributed to their increased risk of congenital anomalies, preterm birth, selective fetal growth restriction, and unique complications of monochorionicity, particularly those related to blood flow imbalance across the monochorionic placenta: twin-to-twin transfusion syndrome (TTTS). TTTS most commonly refers to the severe phenotype, twin oligohydramnios polyhydramnios sequence (TOPS), caused by high blood volume intertwin transfusion. Low flow intertwin transfusion causes twin anaemia-polycythaemia sequence (TAPS). Overall, TTTS affects approximately 15% of all monochorionic twins [4].

TTTS occurs due to the presence of unbalanced vascular anastomoses across the monochorionic placenta, resulting in net blood flow from the ‘donor’ to the ‘recipient’ twin [5]. Twin oligohydramnios polyhydramnios sequence (TOPS) becomes clinically evident between 16 and 24 weeks’ gestation and diagnosis is based on ultrasonographic criteria [6]; the most widely used is the Quintero staging system [7]. The donor twin becomes hypovolaemic, oligohydramnios occurs as urine output diminishes, and growth restriction ensues [8], with Doppler changes consistent with severe placental insufficiency. The recipient twin experiences cardiac overload which can progress to cardiomegaly, myocardial hypertrophy, acquired pulmonary valve stenosis, and hydrops [8]. To re-establish homeostasis, polyuria and polyhydramnios results. Mortality from severe untreated TTTS is high, with reported rates of 70% to 100% [9, 10]. A major contributor to perinatal morbidity and mortality is preterm birth, where untreated TTTS and severe polyhydramnios trigger preterm rupture of the membranes or premature labour. Without treatment, many live births succumb to postnatal complications of TTTS, particularly those related to prematurity, cardiac and renal dysfunction, or complications of polycythaemia and anaemia [9, 11].

The only definitive treatment for TTTS is fetoscopic laser coagulation of placental anastomoses [12]. This reverses the pathological process in TTTS by physically disrupting the anastomosing vessels within the placenta [13]. In 2004, a randomised controlled trial comparing laser surgery with serial amnioreductions demonstrated the clear superiority of laser in terms of survival and survival without major disability [14]. Using a minimally invasive technique, laser surgery is now the first-line treatment for all but the mildest cases of TTTS. Fetoscopic laser coagulation, however, is not without risk. Preterm labour or preterm rupture of membranes occurs in up to 40% of pregnancies due to instrumentation [15, 16]. In up to 18%, surgical failure occurs, resulting in residual anastomoses, low volume ongoing intertwin transfusion, recurrence of TOPS or the emergence of TAPS [6]. A small proportion results in fetal demise*,* donors being at greater risk [17, 18].

Even in the absence of TTTS, the incidence of severe neurodevelopmental abnormalities in preterm monochorionic twins is between 4 and 8% [19]. TTTS increases this background risk of atypical neurodevelopment in survivors. Historically, rates of neurological disability documented among survivors ranged from 17% [11] to 42% [20]. Neurodisability rates improved following the adoption of fetoscopic laser coagulation, with reported rates in survivors of 4% [21, 22] to 31% [23], but study findings have varied on the rigour of the assessment and, particularly, on the length of follow-up which has commonly ceased at 2 years of age. Studies with follow-up to school age are more difficult and costly, but are crucial, as studies with longer durations of follow-up consistently report higher rates of neurodisability [23, 24].

This study aimed to ascertain the presence of long-term neurodevelopmental disabilities in child survivors of TTTS-affected multiple pregnancies managed by placental laser photocoagulation since commencement of a fetal surgery service in 2005.

## Methods

### Study type, setting, and ethics

This observational study of outcomes from the Victorian Fetal Therapy Service was conducted within The Melbourne Children’s campus in Melbourne, Australia. The Victorian Fetal Therapy Service was established in 2005 as a collaboration between the three tertiary obstetric hospitals in the Australian state of Victoria: The Royal Women's Hospital, Monash Health, and Mercy Hospital for Women. Between 2006 and 2017, photocoagulation was undertaken in 120 pregnancies, all performed at Monash Health to centralise experience, and with two or more of the six members of the Victorian Fetal Therapy Service in attendance.

Ethics approval for the study was obtained from the Human Research Ethics Committees (HRECs) of the Royal Children’s Hospital (34269D), Monash Health (RES-17–0000-149X), Mercy Hospital for Women (R15/24) and The Royal Women’s Hospital (HREC/15/RCHM/37). Written or verbal informed consent was obtained from each child’s parent or legal guardian, separate consents being completed for each participating child. The verbal consent procedure was approved by each of the named ethics committees.

### Recruitment of participants

The Victorian Fetal Therapy Service provided a list of the women who had undergone placental laser photocoagulation for TTTS between 2006 and 2017. Initial contact was in the form of a letter to women at their last known address, informing them that they would receive a follow-up phone call after two weeks. Checks of birth and paediatric medical records were made to ensure that study personnel did not knowingly contact families with no surviving children. One of two study personnel (CB or AG) phoned women about the study and sought consent to participate in a telephone interview. Multiple attempts were made to contact women by phone, text message, email, and social media.

### Data collection and outcome measures

During the structured telephone interview, information pertaining to each surviving child was collected from the parent on demographics, plurality, birth gestation, donor/recipient status, co-multiple status, neonatal history, education, medications, medical conditions, developmental concerns, surgeries, health care provision, gross and fine motor function, expressive and receptive language, behaviour, and social skills. During the interview, mothers were asked for consent to complete the Child Behaviour Checklist [25] for each surviving child. Clinical notes and interview responses were recorded using the Research Electronic Data Capture (REDCap) tool [26, 27].

The interviewers evaluated the neurological status of each child based on their history of neurological problems or functional impairment. This included, but was not restricted to, the interviewer’s assessment of whether the information gained from the interview was consistent with a diagnosis of 14 neurodevelopmental conditions: attention deficit (hyperactivity) disorder, autism spectrum disorder, cerebral palsy, epilepsy or seizure disorder, fine motor impairment, genetic abnormality, global developmental delay, intellectual disability, learning disability, myopathy, neural tube defect, sensory impairment, and speech or language delay/disorder.

The Child Behaviour Checklist is a parent/carer-completed instrument to identify emotional and behavioural difficulties in children and adolescents. The questionnaire takes approximately 15 min to complete, and responses are measured on Likert scales. Paper and online versions are available for younger (1.5–5 years) and older (6–18 years) children. The tool produces percentile rankings for specific clusters of difficulty—for example, internalising and externalising behaviours, and subsets of each. The Checklist is a useful and efficient screening tool with well-established validity and reliability [25]. According to a 2008 systematic review, the Child Behaviour Checklist has a sensitivity of 66% [95% CI 60%, 73%] and specificity of 83% [95% CI 81%, 85%] for total problems. Pooled sensitivity varied somewhat across diagnostic domains but estimates of specificity were similar [28].

A three-tiered outcome measure was allocated for each child, consisting of (1) unimpaired or developmentally normal, (2) mild or moderate neurological impairment, or (3) severe neurological impairment. A finding of mild-moderate neurological impairment was based on any reported neurological findings not resulting in severe functional impairment and neurodevelopmental delay, or disability in one or more domain/s on the Child Behaviour Checklist that was objectively mild-moderate and did not result in severe functional impairment. A finding of severe neurological impairment was made the same way but was based on objective evidence of severe functional impairment. Examples of severe functional impairment included cerebral palsy without independent ambulation, severe visual impairment, an Intelligence Quotient (IQ) below 50, and moderate-severe autism spectrum disorder. A follow-up phone call was made to the primary carer to determine whether all appropriate referrals had been made and services were in place. In the few cases where the child was not receiving support, advice was given about how to access this.

### Statistical analysis

Distributions for the grouped year of procedure, plurality, and survival were described for the laser surgery cohort. For study participants, variables from the structured interview were categorised and tabulated. Depending on the plotted distribution of data, means [SD] or medians [range] were calculated for numerical data, including age, birth gestation, length of stay in neonatal intensive care or special care nursery and ventilation time. The 50^th^ centile was used to dichotomise the gestation when laser therapy was performed as < 20 or 20 + weeks. Factors potentially contributing to the risk of neurodevelopmental impairment were assessed using univariable logistic regression analyses with the result reported as odds ratios (OR) and the 95% confidence interval (CI) around each estimate. Multivariable logistic regression was used to adjust for the potential confounding effects of gestation at birth and sex. Mean scores for domains on the Child Behaviour Checklist were calculated. Analysis was performed using Stata software (StataCorp. 2019. *Stata Statistical Software: Release 16*. College Station, TX: StataCorp LLC).

## Results

### The laser surgery cohort

A mean of 10 pregnant women per annum underwent placental laser photocoagulation in Victoria between 2006 and 2017, with little variation in numbers over time. Of the 120 pregnancies, 117 were twin pregnancies, and 3 were triplet. The outcome of 4 twin pregnancies was unknown. Excluding these, 57/113 (50.4%) twin pregnancies resulted in both twins surviving to follow-up (114 survivors), 36 (31.9%) in 1 surviving twin (36 survivors), and 20 (17.7%) in no survivors. One triplet pregnancy resulted in 3 survivors and the other two resulted in 1 survivor (total of 5 survivors). The proportion of pregnancies known to have resulted in at least one surviving fetus at follow-up was 96/116 (83%).

Outcomes were unknown for 8 of the 243 fetuses. Of the 235 fetuses with known outcomes, 154 (65.5%) survived to follow-up. We observed an increase in survival over time, from 59% in the 2006–2008 triennium to 73% in 2015–2017. All but two deaths of co-multiples had occurred antenatally. The sole neonatal co-multiple death occurred in a recipient with an antenatal Grade IV intraventricular haemorrhage, and the sole infant death was related to polymicrogyria in a donor twin.

### Establishing the neurodevelopmental outcomes cohort

**Fig. 1:**
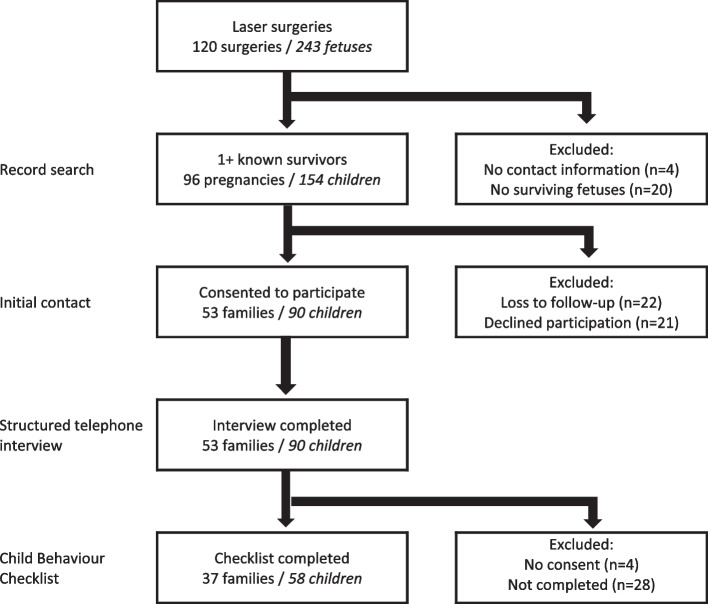
Flow diagram

A flow chart shows our progress throughout the study (Fig. 1). Of the 120 pregnancies, the record search failed to find contact or outcome information for 4 cases, and 20 pregnancies were found to have resulted in no liveborn children. Based on their last known contact details, we attempted to contact the 96 families that were known to have at least one surviving child. Despite multiple attempts, 22 families were uncontactable. Of the 74 families able to be contacted, 21 families declined to participate, and 53 agreed to participate in a telephone interview about the developmental outcomes of their 90 surviving children. This produced a family participation rate of 71.6% (53/74) of those contacted and 55% (53/96) of those that underwent placental laser photocoagulation and had surviving children. The loss-to-follow-up rate in survivors was 41.6%.

### Participant children

Characteristics of the participant children are shown in Table 1. Their mean age was 7.5 [SD 3.0] years at interview. Amongst the 90 participants, 74 were sets of twins (14 male, 23 female twin pairs) and 16 were sole survivors (11 males, 5 females). One sole survivor was from a triplet pregnancy, the surviving triplet being the bystander.

**Table 1:** Characteristics of 90 participating child survivors

	n (%)
Maternal highest education level	
Postgraduate qualification	14 (15.9)
Bachelor’s degree	24 (27.3)
Diploma/certificate	40 (45.4)
Secondary school	10 (11.4)
*Missing*	2
Plurality	
Twins	89 (98.9)
Triplets	1 (1.1)
Sex	
Female	51 (56.7)
Male	39 (43.3)
Donor/recipient status	
Donor	36 (40.0)
Recipient	48 (53.3)
Bystander triplet	1 (1.1)
Reverse	5 (5.6)
Gestation of laser (weeks)	
< 20	40 (44.4)
20 +	50 (55.6)
Year of laser	
2006–8	11 (12.2)
2009–11	24 (26.7)
2012–14	25 (27.8)
2015–17	30 (33.3)
Birth gestation	
37 + weeks	0 (0.0)
32–36 weeks	48 (53.3)
28–31 weeks	37 (41.1)
< 28 weeks	5 (5.6)
Time in special care	
Not admitted	2 (2.2)
< 1 week	3(3.3)
1–4 weeks	30 (33.3)
4–12 weeks	36 (40.0)
> 12 weeks	19 (21.1)
Ventilation time	
None needed/unsure	39 (43.3)
< 1 week	31 (34.4)
1–4 weeks	7 (7.8)
4–12 weeks	7 (7.8)
> 12 weeks	6 (6.7)
Major congenital anomalies	
None	79 (87.8)
One or more	11 (12.2)
Co-multiple survival	
Co-twin survived	74 (82.2)
Co-twin died	15 (16.7)
Co-triplets died	1 (1.1)
Timing of co-multiple demise	
Antenatal	14 (87.5)
Neonatal	1 (6.2)
Postneonatal	1 (6.2)
*Not disclosed*	*1*
Age at interview	
2.5–4.9 years	22 (24.4)
5.0–9.9 years	46 (51.1)
10 + years	22 (24.4)

All participant children were born between 25 and 36 weeks’ gestation (mean 31.8 [SD 2.7]). Of the 90 children, only two were not admitted to a neonatal special care nursery. The remaining 88 children spent a median time of 39.5 days (range 1–183) in special care. Respiratory support in terms of intubation/ventilation or continuous positive airway pressure (CPAP) was required for 51 (56.7%) children for a median period of 4 days (range 1–94).

Congenital anomalies were identified in 11/90 children, including 2 sets of twins. Anomalies comprised a perimembranous ventricular septal defect (1), atrial septal defect (1), pulmonary valve stenosis (4), hypospadias (3), craniosynostosis (1), polycystic kidney (1), talipes (1), and reduction defect of upper limb (1). In all 4 children with pulmonary stenosis, the condition was diagnosed post laser surgery, 2 antenatally and 2 neonatally. All were recipients at the time of surgery. At least one congenital anomaly was identified in 6/36 donors and 4/48 recipients.

### Neurodevelopmental outcomes

#### Parental interview

Based on information obtained from the comprehensive parental interview and medical record search, 58/90 (64%) children were deemed to have made developmental progress that was within normal limits, 20 (22%) had diagnosed neurological or developmental conditions, and another 12 (13%) had equivocal or uncertain findings (Table 2). Some children had more than one condition. Speech and language disorders were the most common finding, followed by attention deficit (hyperactivity) disorder and fine motor impairment (Table 2). Strong evidence for a sex difference was only seen for attention deficit (hyperactive) disorder; 1/51 (2%) girls and 8/39 (20%) boys had previously received this diagnosis.

**Table 2:** Neurodevelopmental diagnoses for 90 child participants

	Number of diagnoses
Speech/language delay/disability	15
Attention deficit (hyperactivity) disorder	10
Fine motor impairment/delay	10
Global developmental delay	8
Autism spectrum disorder	7
Sensory impairment	6
Learning disability	5
Genetic abnormality	2
Tic disorder	2
Cerebral palsy	1
Metabolic disorder	1
Epilepsy or seizure disorder	0
Intellectual disability	0
Myopathy	0
Neural tube defect	0

#### Child Behaviour Checklist

One family declined to complete the Child Behaviour Checklist, and another did not complete it due to a significant language barrier. Of the 51 families agreeing to complete the questionnaire on behalf of their 86 children, 37 families returned 58 responses, 41 in the 6–18 age bracket and 17 under age 5. Based on percentile scores in the atypical range for relevant item subsets on the Checklist, all previous diagnoses were confirmed. No new difficulties or potential diagnoses were identified. Compared to two Australian normative samples [29, 30], higher mean scores on the age 6–12 version of the Child Behaviour Checklist indicated more problems on most domains in boys but not in girls (Table 3).

**Table 3:** Mean scores on the Child Behaviour Checklist (CBCL) for the TTTS and laser surgery cohort compared to two local control samples

	TTTS	Control 1^a^	Control 2^a^	TTTS	Control 1	Control 2
	Boys 6–13 *n* = 23	Boys 7 *n* = 333	Boys 6–11 *n* = 300	Girls 6–13 *n* = 18	Girls 7 *n* = 231	Girls 6–11 *n* = 300
CBCL domain	Mean [SD]	Mean [SD]	Mean [SD]	Mean [SD]	Mean [SD]	Mean [SD]
Activities	9.1 [3.2]	6.9 [2.1]	7.6 [2.0]	9.5 [3.2]	7.3 [2.0]	8.3 [1.8]
Social	7.6 [2.9]	5.9 [1.7]	6.5 [1.9]	6.0 [2.7]	6.0 [1.7]	6.3 [1.8]
School	4.7 [1.0]	5.0 [0.8]	4.0 [0.9]	4.0 [1.4]	5.1 [0.7]	5.3 [0.8]
Competence	21.3 [6.0]	17.9 [3.2]	18.6 [3.3]	19.5 [5.3]	18.4 [3.1]	20.0 [3.1]
Internalizing	8.9 [9.4]	6.4 [5.3]	8.5 [6.3]	8.8 [7.9]	7.6 [6.3]	9.0 [5.5]
Externalizing	10.2 [12.3]	9.3 [6.6]	12.4 [7.5]	5.1 [7.5]	7.3 [6.3]	10.4 [6.2]
Problems	37.7 [39.7]	24.6 [15.4]	32.4 [17.7]	27.9 [27.1]	23.2 [17.0]	30.6 [15.0]

^a^Control group 1 was randomly sampled from schools in metropolitan Melbourne, Australia, and excluded children seen by a psychologist or psychiatrist in the previous year [30]. Approximately half of the sample had asthma. Control group 2 was a non-selected normative sample from Sydney, Australia [29]

#### Overall neurodevelopmental status and clinical predictors

Based on information gained from the parental interview and supported by additional evidence from the Child Behaviour Checklist, 28/90 (31%) children were deemed to have neurodevelopmental impairment, 27 of mild-moderate severity and 1 severe. Length of stay in neonatal higher care conferred the highest risk of impairment, 7.6 times the risk for 4–12 weeks relative to 0–4 weeks [95% CI 1.96, 29.57], and 11.8 times the risk for more than 12 weeks [95% CI 2.68, 52.44] (Table 4). Statistical adjustment for sex and grouped gestation at delivery decreased the odds to 6.4 [95% CI 1.6, 26.7] and 8.4 [95% CI 1.6, 45.1], respectively. There was also strong evidence for increased risk of impairment for delivery at 25–31 weeks’ gestation (OR 3.58 [95% CI 1.39, 9.22]), assisted ventilation (OR 3.79 [95% CI 1.35, 10.64]), and lower levels of maternal education (OR 3.21 [95% CI 1.19, 8.66]; Table 4). When adjusted for sex and gestation at birth, there remained only weak evidence for the contribution to the risk of neurological impairment of assisted ventilation, whereas adjustment did not affect the risk conferred by lower levels of maternal education.

**Table 4:** Univariable analysis of risk factors for neurodevelopmental impairment

	Neurodevelopment		Odds ratio [95% CI]	*p*-value
	Unimpaired (*n* = 62) n (row %)	Impaired (*n* = 28) n (row %)		
Maternal highest education level				
Postgraduate/Bachelor	31 (81.6)	7 (18.4)	Reference	Reference
Diploma/certificate/secondary	29 (58.0)	21 (42.0)	3.21 [1.19, 8.66]	0.022
Sex				
Female	36 (70.6)	15 (29.4)	Reference	Reference
Male	26 (66.7)	13 (33.3)	1.20 [0.49, 2.94]	0.691
Child’s age at interview (years)				
0–4.9	18 (81.8)	4 (18.2)	Reference	Reference
5–9.9	30 (65.2)	16 (34.8)	2.4 [0.69, 8.31]	0.167
10 +	14 (63.6)	8 (36.4)	2.6 [0.64, 10.31]	0.183
Donor/recipient status				
Donor	22 (61.1)	14 (38.9)	Reference	Reference
Recipient	34 (70.8)	14 (29.2)	0.65 [0.26, 1.61]	0.351
Other	6 (100.0)	0 (0.0)	n/a	n/a
Gestation of laser (weeks)				
< 20	30 (75.0)	10 (25.0)	Reference	Reference
20 +	32 (64.0)	18 (36.0)	1.69 [0.67, 4.23]	0.265
Year of laser				
2006–8	8 (72.7)	3 (27.3)	Reference	Reference
2009–11	13 (54.2)	11 (45.8)	2.26 [0.47, 10.64]	0.304
2012–14	17 (68.0)	8 (32.0)	1.25 [0.26, 6.03]	0.777
2015–17	24 (80.0)	6 (20.0)	0.67 [0.13, 3.30]	0.619
Birth gestation				
32–36 weeks	39 (81.2)	9 (18.8)	Reference	Reference
25–31 weeks	23 (54.8)	19 (45.2)	3.58 [1.39, 9.22]	0.008
Time in special care				
< 4 weeks	32 (91.4)	3 (8.57)	Reference	Reference
4–12 weeks	21 (58.3)	15 (41.7)	7.62 [1.96, 29.57]	0.003
> 12 weeks	9 (47.4)	10 (52.6)	11.85 [2.68, 52.44]	0.001
Ventilation required				
No	31 (83.8)	6 (16.2)	Reference	Reference
Yes	30 (57.7)	22 (42.3)	3.79 [1.35, 10.64]	0.011
Co-multiple survival				
Sole survivor	12 (75.0)	4 (25.0)	Reference	Reference
Co-twin alive	50 (67.6)	24 (32.4)	1.44 [0.42, 4.94]	0.562
Congenital anomalies				
No	56 (70.9)	23 (29.1)	Reference	Reference
Yes	6 (54.6)	5 (45.4)	2.03 [0.56, 7.31]	0.279

## Discussion

In this observational study, we evaluated the neurodevelopmental status of 90 surviving children following placental laser coagulation for TTTS performed in the Australian state of Victoria between 2006 and 2017. Twenty-eight (31%) children were assessed as having neurodevelopmental impairment, with only one being severe. To provide context on the risk ‘above backround’ associated with TTTS requiring laser treatment, Australian population data show that 7.7% of children under the age of 15 years have a disability [31]. Two methods were used to inform a composite measure for neurodevelopmental outcomes. First, we systematically collected information via a comprehensive parental phone interview and medical record review, and many parents completed the Child Behaviour Checklist for each surviving child. This study provides parents contemplating this procedure access to information on the long-term developmental outcomes to help inform decision-making about the pregnancy and to help promote better adjustment to parenthood and developmental surveillance [32]. It informs future research on the biological and clinical information needed to disentangle the factors on the pathway to a developmental disability for children treated with laser surgery for TTTS. Importantly, these local data underscore the importance of long-term follow-up of this vulnerable group of children.

It was difficult to compare our rate of neurodevelopmental impairment with rates in the published literature, in part because rates varied markedly depending on the assessment tools used, length of follow-up, and the types and definitions of outcomes included. We aimed to include a broad description of neurodevelopmental impairment, with speech and language disorders, attention deficit (hyperactivity) disorder, and fine motor impairment identified as the most common types. The findings of a 2020 systematic review of 24 studies included a mean prevalence of 5% for cerebral palsy compared to 1 in 90 (1%) in our study, and a mean prevalence of 10.5% for any neurodevelopmental impairment compared to our 31% [24]. Although most of the reviewed studies assessed for a limited number of impairments, Müllers et al.reported a rate of 14.2% for neurodevelopmental impairment at a median age of four years that included cerebral palsy, speech and language delay, behavioural concerns such as autism, and mild motor delay [33]. The prevalence closest to our estimate of 31% was reported in a study by Tosello et al.that used scores derived from the Ages and Stages Questionnaire and clinical examination to arrive at a prevalence of 31.4% for children at a mean age of 37 months [23]. The proportion of children with neurodevelopmental impairment in our study was also similar to that reported from a recent study undertaken in The Netherlands, where the rate of impairment was 46% at 5 years, and impairment severity was predominantly mild-moderate [34]. The Dutch estimate arose from routine follow-up of the subgroup of child survivors born before 30 weeks’ gestation or small for gestational age, so may not be generalisable to all survivors.

Knijnenburg et al., authors of the 2020 systematic review cited above, recognised that an important limitation of comparison between studies was the substantial difference in length of follow-up, ranging from 1 month to 10 years in the reviewed studies [24]. The review identified probable under-ascertainment of neurodevelopmental impairment in children under the age of five years, a finding supported in their subsequent local study [34]. Similarly, we found the rate of neurodevelopmental impairments in children under 5 was half that of those aged 5 and older. In our study, 75% of children were 5 years or older, with the mean age at follow-up 7.5 years. Only 15 of the 90 children were under 4.5 years of age. The combination of greater maturity at follow-up and the comprehensive list of neurodevelopmental inclusions in the Dutch and our Australian study may explain the higher rates of impairment in both studies.

We were able to associate the risk of long-term neurodevelopmental impairment in children that survived placental laser photocoagulation with maturity at delivery and neonatal wellbeing. Early birth gestation and low birthweight were similarly recognised as risk factors in studies included in two systematic reviews, the Knijnenburg et al.review [24], and a 2021 review by Hessami et al. [35]. Higher Quintero TTTS stage at laser was determined to increase risk in 4 of 5 studies included in the Knijnenburg et al.review but was not significantly associated with neurodevelopmental impairment in the Hessami et al.review. We were unable to include Quintero stage in the present study, but, in the first 5 years of the Victorian service, there was no significant difference in perinatal survival by Quintero stage (77% for stages 2–3 versus 66% for stage 4) [36]. We found little evidence to support a difference in long-term impairment between donor and recipient twins. This finding was supported by data from three systematic reviews [18, 24, 35]. There were mixed findings in the Knujnenburg et al.systematic review with respect to maternal level of education [24]. In our study, there was strong evidence in support of a higher frequency of neurodevelopmental impairment in children of mothers who had not completed a bachelor’s degree or higher.

Our relatively small sample size necessitated the use of broader groups for risk factor analysis and limited confidence around the results. Even with larger numbers, it is not easy to disentangle the confounding effects on neurodevelopmental outcomes of plurality, chorionicity, fetal growth and weight discordance, birth gestation, impact of fetoscopic laser coagulation, and family factors. Twins are more likely to be born prematurely, triplets even more so, and there is likely to be an additional contribution of monochorionic-specific complications [3]. Preterm birth increases the risk of speech and language disorders [37] and attention deficit (hyperactivity) disorder [38], the two most common neurodevelopmental impairments in our study. A range of control groups and very large samples would be needed to better understand the contribution of each factor. Although we were able to compare mean scores on the Child Behaviour Checklist to two published control groups [29, 30], we did not have the resources to recruit one or more control groups specifically for the study. Similarly, only four studies in the recent systematic review compared the neurodevelopmental outcomes of TTTS survivors with a control group [24], and 3 different control groups were employed. Based on these four studies, the rate of neurodevelopmental impairment in survivors of laser photocoagulation therapy for TTTS was higher than for uncomplicated singletons [39], comparable to uncomplicated monochorionic twins when adjusted for birth gestation, birthweight, and gender [40], and comparable to dichorionic twins matched for birth gestation [41, 42].

Long-term follow-up of all monochorionic pregnancies is vital. Not only is this information important for family counselling and decision-making, but it would facilitate monitoring progress in preventing morbidity and enable the use of uncomplicated monochorionic twins as an appropriate control group for assessing outcomes. In the future, rates of spontaneous preterm birth might be reduced with improved surgical techniques to lower the risk of procedure-related complications and preterm rupture of membranes. We report a gradual improvement in surgical outcomes over time in this cohort, although small numbers mean the improvement may have been slower than that seen in higher volume centres [43]. Improvement in surgical outcomes across time is likely to be multifactorial, including (i) increased surgical experience, (ii) adoption of advances of surgical technique (such as Solomisation of the vascular equator), and (iii) technical advances resulting in reduced calibre of the fetoscopic ports and improved in utero visualisation with the advent of 0^O^ scopes [43]. Going forward, the rates of iatrogenic preterm delivery might be lowered by the implementation of new therapies for complications leading to fetal deterioration, such as severe growth restriction and Twin Anaemia Polycythaemia Sequence [44]. To optimise outcomes, follow-up after delivery would enable early intervention from multi-disciplinary teams and might provide evidence to highlight the special social and economic needs of these families [44].

Loss to follow-up was a limitation in our study, particularly since most studies show that the infants most likely to be impaired are those least likely to return for follow-up. Only 90 of 154 survivors were included, thereby creating a source of bias and the potential for under-reporting of moderate-severe neurodisability. Revisiting difficult emotions and the time required for assessments were important reasons, the latter exacerbated by multiple extended lockdowns during the Covid 19 pandemic. Nevertheless, this study demonstrates the feasibility of accessing informative data through telehealth. Although children did not undergo direct neurological evaluation for the study, they had been assessed by other health professionals and we are cautiously confident in our assessment of neurodevelopmental impairment. Limitations created by our relatively small sample size and lack of one or more comparison groups have been discussed above.

## Conclusion

Substantial variation exists between fetal therapy services in the type and length of neonatal follow-up for monochorionic pregnancies and fetoscopic laser coagulation [44]. This contributes to a lack of available data with which families can make informed decisions regarding care. There is a paucity of studies that report school age outcomes of TTTS survivors, yet these outcomes are of great importance to families and to those caring for them [44, 45]. The findings from this study support the need for systematic and standardized follow-up of survivors of TTTS and demonstrate the potential for telemedicine to be used alone or in combination with in-person developmental assessment. This may improve equity of access to follow-up, and thus uptake. Routine long-term follow-up will better inform the need for targeted intervention of vulnerable infants, to optimize neurodevelopmental outcomes.

## Data Availability

The datasets used and/or analysed during the current study are available from the corresponding author on reasonable request.
